# Speech compensation responses and sensorimotor adaptation to formant feedback perturbations

**DOI:** 10.1121/10.0003440

**Published:** 2021-02-17

**Authors:** Inez Raharjo, Hardik Kothare, Srikantan S. Nagarajan, John F. Houde

**Affiliations:** 1 University of California, Berkeley and University of California, San Francisco, Graduate Program in Bioengineering; 2 Biomagnetic Imaging Laboratory, Department of Radiology and Biomedical Imaging, University of California San Francisco, San Francisco, California 94143, USA; 3 Speech Neuroscience Laboratory, Department of Otolaryngology—Head and Neck Surgery, University of California San Francisco, San Francisco, California 94143, USA

## Abstract

Control of speech formants is important for the production of distinguishable speech sounds and is achieved with both feedback and learned feedforward control. However, it is unclear whether the learning of feedforward control involves the mechanisms of feedback control. Speakers have been shown to compensate for unpredictable transient mid-utterance perturbations of pitch and loudness feedback, demonstrating online feedback control of these speech features. To determine whether similar feedback control mechanisms exist in the production of formants, responses to unpredictable vowel formant feedback perturbations were examined. Results showed similar within-trial compensatory responses to formant perturbations that were presented at utterance onset and mid-utterance. The relationship between online feedback compensation to unpredictable formant perturbations and sensorimotor adaptation to consistent formant perturbations was further examined. Within-trial online compensation responses were not correlated with across-trial sensorimotor adaptation. A detailed analysis of within-trial time course dynamics across trials during sensorimotor adaptation revealed that across-trial sensorimotor adaptation responses did not result from an incorporation of within-trial compensation response. These findings suggest that online feedback compensation and sensorimotor adaptation are governed by distinct neural mechanisms. These findings have important implications for models of speech motor control in terms of how feedback and feedforward control mechanisms are implemented.

## I. INTRODUCTION

Speaking is unique among motor behaviors for human communication—it is the prime conveyor of linguistic intent—and it depends upon incredibly precise timing and coordination of many independent articulators. The most basic defining feature of speaking that sets it apart from other motor actions is that it produces sound. Yet in spite of its importance to speech, the role of auditory feedback in normal speech production and in the control of speech remains unclear. One plausible way for speech to be maintained and controlled is through the monitoring of auditory feedback and using that feedback information to correct for errors in speech output. Although the sensory processing delays (50–150 ms), seen in human and non-human primates (Bendor and Wang, 2005; Houde and Nagarajan, 2011; Jenkins *et al.*, 2010; Poeppel and Hickok, 2015), inherent to this correction process rule out the possibility that speech is controlled solely and directly by auditory feedback, research has shown that the control of natural speech is nevertheless responsive to changes in auditory feedback (Lametti *et al.*, 2018; Liu *et al.*, 2009). Auditory feedback thus plays a modulatory role in ongoing speech production, even in dynamically changing natural speech. Numerous studies have investigated the role of auditory feedback in speech production through various auditory feedback perturbation experiments, which usually examine responses to either unpredictable or consistent, predictable perturbations (Burnett *et al.*, 1998; Caudrelier and Rochet-Capellan, 2019, pp. 15–75; Chen *et al.*, 2007; Houde and Jordan, 1998; Houde and Nagarajan, 2015, pp. 267–298).

Within-trial online compensation has been observed in response to unpredictable auditory feedback perturbations, i.e., perturbations that are randomly and unexpectedly applied within an utterance. Speech production models (Guenther, 2016; Houde and Nagarajan, 2011; Kearney *et al.*, 2020; Parrell *et al.*, 2019) have theorized that within-trial online compensatory responses are generated by a feedback control mechanism, where auditory feedback is compared to an internal representation of expected auditory feedback. If a mismatch is detected, motor correction commands are sent to speech articulators to correct for the mismatch. Studies have investigated the within-trial online compensatory responses to unpredictable pitch and loudness perturbations, both at mid-utterance and at utterance onset (Bauer *et al.*, 2006; Burnett *et al.*, 1998; Hawco and Jones, 2009; Heinks-Maldonado and Houde, 2005; Keough *et al.*, 2013; Larson *et al.*, 2001; Larson *et al.*, 2000; Larson *et al.*, 2007; Patel *et al.*, 2015; Scheerer and Jones, 2018). In the control of pitch, some differences have been observed in online compensatory responses to pitch feedback perturbations that occur at mid-utterance or at utterance onset in the same set of subjects, suggesting possible different mechanisms governing control of pitch at onset and at mid-utterance (Hawco and Jones, 2009; Scheerer and Jones, 2018). In the control of formants, responses to unpredictable perturbations applied at utterance onset have been studied (Cai *et al.*, 2012; Niziolek and Guenther, 2013; Reilly and Dougherty, 2013; Tourville *et al.*, 2008). In general, these studies have used digital filtering techniques to directly alter formants in subjects' speech feedback at utterance onset and have found a short-latency compensatory response occurring ∼150 ms after utterance onset. Only one study has investigated the responses to unpredictable formant feedback perturbations at mid-utterance: Purcell and Munhall (2006b) studied responses to *F*1 perturbations that began 300 ms after speech onset and were gradually introduced to full strength over 500 ms. They found a compensatory response occurring ∼450 ms after perturbation onset. It is important to note, however, that this gradual perturbation onset technique is not directly comparable to the sudden introduction of perturbed feedback used in most other feedback perturbation studies. A study of formant perturbations at mid-utterance that is more comparable with the aforementioned studies of formant perturbations at utterance onset is called for. Moreover, it would be desirable for a study to look at identical unpredictable perturbations applied at mid-utterance or at utterance onset in the same set of subjects. Lacking such a study, it remains unclear whether shared online compensation mechanisms exist for formant control at utterance onset and at mid-utterance.

Understanding the mechanisms of within-trial compensatory responses would help us determine how these mechanisms relate to another type of speech response to perturbed feedback—across-trial sensorimotor adaptation. Across-trial sensorimotor adaptation has been observed in the production of both pitch and formants (Houde and Jordan, 1998; Jones and Munhall, 2000; Katseff *et al.*, 2012; Purcell and Munhall, 2006a) in response to consistent, predictable auditory feedback perturbations, i.e., perturbations that are consistently applied over many trials have been observed. There is a general agreement among speech production models (Guenther, 2016; Houde and Nagarajan, 2011; Kearney *et al.*, 2020; Parrell *et al.*, 2019) that this sensorimotor adaptation involves learning long-term changes in feedforward control that gradually anticipate the effects of consistent, predictable auditory feedback perturbations. The mechanism that accomplishes this sensorimotor adaptation of feedforward control, however, is less clear. The directions into velocities of articulators (DIVA) model and its simpler version, the SimpleDIVA model, assume a close relationship between feedback and feedforward control, wherein sensorimotor adaptation arises from feedforward control being learned via incorporation of online feedback compensations (Guenther, 2016; Kearney *et al.*, 2020). As an alternative DIVA, state feedback control (SFC) models have long been used in non-speech motor behaviors (Shadmehr *et al.*, 2010), and more recently have also been applied to speech behaviors (Houde and Nagarajan, 2011; Parrell *et al.*, 2019). SFC models, on the other hand, can accommodate adaptation resulting directly from sensory prediction errors rather than necessarily being derived from the incorporation of corrective movements as is assumed in DIVA. Data consistent with SFC models have shown that online compensation and sensorimotor adaptation can be differently affected when comparing patient and control groups (Abur *et al.*, 2018; Chen *et al.*, 2013; Demopoulos *et al.*, 2018; Mollaei *et al.*, 2016; Mollaei *et al.*, 2013; Parrell *et al.*, 2017), suggesting a potential underlying difference between the control mechanisms of online compensation and sensorimotor adaptation. Given that both online compensation and sensorimotor adaptation responses are observed in the production of formants, a question arises as to whether both are controlled with the same underlying neural mechanism.

The current study investigated the within-trial online compensation and across-trial sensorimotor adaptation responses to formant feedback perturbations during speaking to elucidate the relationship between the feedback and feedforward control mechanisms of speech. We first examined online compensation responses to two types of unpredictable formant feedback perturbations: (1) whole-trial perturbations applied at utterance onset, and (2) transient perturbations applied at mid-utterance. We applied these perturbations at varying magnitudes and directions, similar to prior feedback perturbation studies (Bauer *et al.*, 2006; Burnett *et al.*, 1997; Cai *et al.*, 2012; Hawco and Jones, 2009; Katseff *et al.*, 2012; Larson *et al.*, 2001; Parrell *et al.*, 2017; Purcell and Munhall, 2006b), to increase the unpredictability of the perturbations and to investigate whether the magnitude and direction of perturbations have an effect on the online compensation responses. Similar to that seen in responses to pitch feedback perturbations, we hypothesized we would see within-trial online compensatory responses to both the utterance-onset and mid-utterance types of formant feedback perturbations. We further examined whether responses to these two types of feedback perturbations are governed by different mechanisms (as has been suggested in Hawco and Jones, 2009) using regression analyses, which has commonly used to study the relationship of speech responses and underlying mechanisms (Lametti *et al.*, 2012; Lametti *et al.*, 2014; Mollaei *et al.*, 2019). If the within-trial online compensatory responses to utterance-onset and mid-utterance types of feedback perturbations are governed by different mechanisms, as has been suggested to be the case for responses to pitch feedback perturbations, we would predict that the responses to formant perturbations at utterance onset and at mid-utterance will not be correlated with each other. Alternately, if these two within-trial online compensatory responses are governed by similar mechanisms, we would expect a correlation between the respective compensatory responses.

We also examined sensorimotor adaptation responses to consistent, predictable formant feedback perturbations. First, we examined the relationship between within-trial online feedback compensation to unpredictable formant perturbations and sensorimotor adaptation to consistent, predictable formant perturbations. If sensorimotor adaptation processes depend on within-trial online feedback compensation processes, we would expect a correlation between within-trial online compensation and across-trial sensorimotor adaptation responses. Alternatively, if they are governed by distinct neural mechanisms, we would predict that sensorimotor adaptation responses will not be correlated with within-trial online compensation responses to either types of unpredictable formant feedback perturbations.

Finally, we investigated the relationship between across-trial sensorimotor adaptation and within-trial online compensation by examining how the within-trial responses evolved during sensorimotor adaptation. If the mechanisms for sensorimotor adaptation depend on within-trial online compensation, we would at the very least expect that throughout the trials of the sensorimotor adaptation, the within-trial online compensation response would gradually be subsumed by a feedforward sensorimotor adaptation response that starts at the beginning of each trial. Alternatively, if online compensation and sensorimotor adaptation are governed by distinct neural mechanisms, then we might expect to see no association between within-trial responses and the growth of across-trial sensorimotor adaptation.

## II. METHODS

### A. Participants

Healthy participants were recruited for the study (*n* = 23, seven females) through UC Berkeley class announcements, pamphlets, and online platforms. Participants' ages ranged from 18 to 43 years (mean ± standard deviation of 21.7 ± 5.5 years). All participants were native English speakers and the majority were bilingual/multilingual (*n* = 22). One multilingual participant's data were taken out because their sensorimotor adaptation response was an outlier that far exceeded three standard deviations from the median sensorimotor adaptation across participants. As a result, data from 22 participants was included in the analysis. Participants had no deficits in learning, motor, or speech and language abilities and gave written informed consent to participate. The study was approved by the University of California, San Francisco (UCSF) Institutional Review Board for human research.

### B. Apparatus

The experiments were performed in a quiet room equipped with sound booth. While inside the sound booth, participants sat in front of a laptop (Thinkpad W530, Lenovo Group Limited) while wearing Beyerdynamic DT 770 Pro 250 Ohm headphones and a head-mounted AKG Pro Audio C520 condenser microphone. Participant's speech from the microphone was fed into a Focusrite Scarlett 2i2 USB Recording Audio Interface and processed and recorded using matlab (which also displayed the word prompts) paired with the Feedback Utility for Speech Production (FUSP) software. FUSP repeatedly analyzed 3 ms frames of speech input from the microphone into separate pitch and formant representations that were, at times, altered (depending on the experiment) and used to synthesize the next 3 ms of speech output to the participant's headphones. The speech data were recorded at a rate of 11 025 Hz and this feedback processing, along with hardware delays, introduced an imperceptible ∼21 ms delay in the auditory feedback, as measured following the methods outlined by Kim *et al.* (2020).

### C. Experimental design and procedures

The current study consisted of five sessions of 165 trials each with formant feedback perturbations. Trials in sessions 1, 3, and 5 included *unpredictable formant perturbations*, applied either at utterance onset or at mid-utterance, performed to examine *within-trial online compensation responses*. Trials in sessions 2 and 4 included *consistent, predictable formant perturbations*, performed to examine *across-trial sensorimotor adaptation responses*. For any given trial, participants were instructed to say either the word “head” (/hɛd/) or “hid” (/hId/), extending the vowel portion of the utterance for as long as the prompt word was displayed on the screen (approximately 2 s). The “hid” catch trials were infrequent (number of trials discussed below). These catch trials were added to (1) keep the experiment more engaging so as to prevent participants from becoming bored and inattentive to the speech production task, and to (2) encourage participants to make contrastive productions of these vowels and pay more attention to the acoustics of their production. Before the first session, a short practice session with experimenter (about 5–10 trials as needed) was done to ensure that the participant could hold out their word steadily as instructed. Within each session, there were random-length breaks (1.7–2.7 s) between trials as well as self-paced breaks after every 15 trials. Five-minute breaks were also administered in between sessions in an attempt to wash out possible carryover effects from previous sessions, during which the experimenter would verbally engage with the participant, for example, by asking questions relating to the current experiment.

**FIG. 1.**
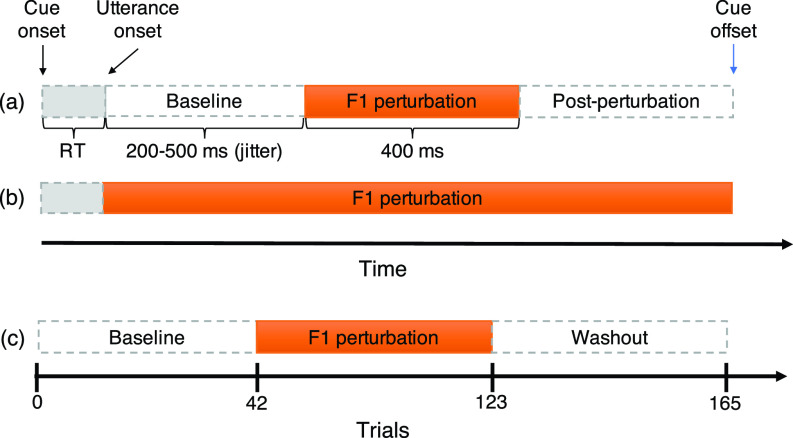
**Experimental paradigm.** *F*1 perturbation applied at different timescales: (a) unpredictable transient mid-utterance perturbation (a 400 ms perturbation initiated after a 200–500 ms jitter delay from utterance onset); (b) unpredictable whole utterance perturbation, initiated at utterance onset and sustained for the whole utterance; and (c) consistent, predictable utterance-onset whole utterance perturbation applied over many trials (sensorimotor adaptation). RT = Reaction time light gray bars with dashed outline). Bars (light gray and white) with dashed outline indicate where no perturbation was applied, and solid orange bars indicate where perturbation was applied.

**FIG. 2.**
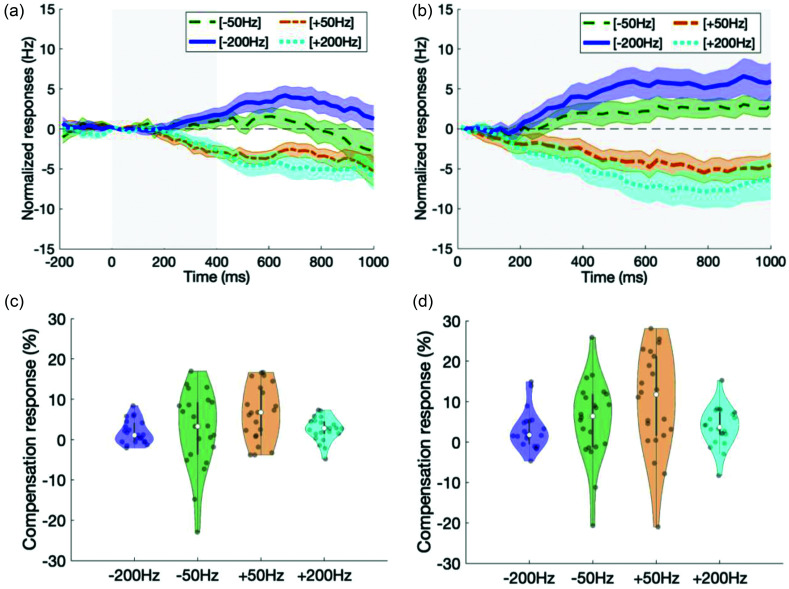
**Online formant response to unpredictable mid-utterance and whole utterance formant perturbations.** Normalized *F*1 responses averaged across participants within each condition are shown for (a) unpredictable transient mid-utterance and (b) unpredictable whole-utterance *F*1 perturbations. Time-range of perturbation are shown as shaded gray area (perturbation onset at *t* = 0). Mean responses (lines) and SEM (shaded colored region) are shown. Responses to −50 Hz perturbations are indicated with dashed line (green), +50 Hz perturbations with dashed-dotted line (orange), −200 Hz perturbations with solid line (blue), and +200 Hz perturbations with dotted line (cyan). The distribution of peak compensation responses for each condition are shown as violin plots for (c) unpredictable transient mid-utterance and (d) unpredictable whole-utterance *F*1 perturbations. The gray bar indicates the range from the 1st to 3rd quartile and the white dot indicates the median. The shape of the violin plot reflects the kernel density estimate of the data, and the colored dots are actual individual response data points. Table I lists the *p-*values for significant differences from zero of the peak compensation responses.

Within-trial online compensation response data were collected from 495 trials evenly distributed across sessions 1, 3, and 5. Session 1 began with 15 familiarization phase trials included to acquaint participants to the experimental task and pace, with each trial being randomly chosen from any one of the trial types described below. The remaining 480 trials were distributed across the remainder of session 1 as well as sessions 3 and 5. These 480 trials consisted of 432 perturbation trials (360 “head” and 72 “hid” trials) and 48 unperturbed trials (30 “head” and 18 “hid” trials). Perturbation trials were unpredictable perturbations of the first formant (*F*1) of subjects' auditory feedback. Two types of unpredictable formant perturbations were applied: (1) unpredictable formant perturbations applied at mid-utterance, transiently for 400 ms with a 200–500 ms jittered delay from utterance onset [*unpredictable mid-utterance perturbations*, Fig. 1(a)], and (2) unpredictable formant perturbations at utterance onset applied for the entire trial duration [*unpredictable whole utterance perturbations*, Fig. 1(b)]. The 400 ms duration for the transient perturbations was chosen as it has often been used in previous mid-utterance pitch and loudness perturbation studies (Heinks-Maldonado and Houde, 2005; Kort *et al.*, 2016). Four different *F*1 feedback perturbations were applied: −50 Hz, +50 Hz, −200 Hz, and +200 Hz for each mid- and whole-utterance perturbation, totaling to eight formant feedback perturbation conditions. These eight conditions were randomly distributed across the perturbation trials (see supplementary Table I).^1^ The choice of perturbation magnitudes of 50 and 200 Hz are well within the range of values that have been used in prior formant sensorimotor adaptation studies (Katseff *et al.*, 2012; MacDonald *et al.*, 2010).

**TABLE I.** Latency and magnitude of responses to unpredictable perturbations for each condition. Response onset latency was calculated from the across-participant averaged responses. Peak compensations across participants (mean and SEM) were calculated in the 200 ms window around the peak latency of the averaged group response. T-values (two-tailed) and *p*-values indicate the significant difference from zero of the peak compensations.

Perturbation type	Perturbation value (Hz)	Onset latency (ms)	Peak compensation (%) (Mean ± SEM)	T-values (degrees of freedom, DoF = 21)	Significance level (*p*-value)
Mid	−50	450	2.36 ± 2.07	1.14	0.27
	+50	200	6.63 ± 1.47	3.97	1.94 × 10^−4^
	−200	275	1.86 ± 0.62	3.01	6.66 × 10^−3^
	+200	275	2.50 ± 0.63	3.97	6.92 × 10^−4^
Whole	−50	625	5.34 ± 2.12	2.52	0.02
	+50	325	9.94 ± 2.66	3.73	1.23 × 10^−3^
	−200	275	2.92 ± 1.02	2.88	9.02 × 10^−3^
	+200	200	3.82 ± 1.03	3.70	1.33 × 10^−3^

Across-trial sensorimotor adaptation response data were collected in session 2 and 4. In each of these sessions, the *F*1 perturbation was consistently applied over many trials arranged as a sequence of phases [Fig. 1(c)]: (1) a baseline phase of 42 trials (30 “head” trials and 12 “hid” trials), where feedback was unperturbed, (2) a hold phase of 81 trials (60 “head” trials and 21 “hid” trials), where *F*1 feedback was consistently perturbed by whole-trial perturbations of either +200 or −200 Hz (depending on the session), and (3) a washout phase of 42 trials (30 “head” trials and 12 “hid” trials), where feedback was again unperturbed. The session order for the two *F*1 perturbation directions was counterbalanced among participants, i.e., half of the participants received +200 Hz *F1* feedback perturbation for session 2 and –200 Hz *F*1 feedback perturbation for session 4; for the other half, this order was reversed. The “hid” trials were again included as catch trials and not included in the analysis. The “head” and “hid” trials were randomized within each phase of each adaptation session.

### D. Data processing and statistical analysis

All acoustic speech data were analyzed using Wave Viewer, a custom-built matlab-based speech analysis software (https://github.com/SpeechNeuroscienceLab/Wave-Viewer) (Houde, 2014). In each trial, formants were tracked using linear predictive coding (LPC). The tracking for the first formant was further refined by manual screening, as needed, to exclude bad trials (e.g., trials with no speech response, interruption in speech production/recording, and poor formant tracking) and to occasionally fix the voice onset and offset time markings automatically detected by FUSP. The analysis focused on all good “head” trials (including those from the familiarization phase). The “hid” trials were excluded from analysis because they were designed as infrequent catch trials and were thus unsuitable for reliable statistical analysis. On average, less than 3% of “head” trials were excluded from analysis across all subjects. Using the raw, *F*1 formant track trial data extracted, the following six analyses were performed.

#### 1. Within-trial online compensation responses to unpredictable formant perturbations

To obtain an *F*1 response time-course that could highlight each participant's *F*1 response changes elicited by the perturbations, we performed three linear normalization steps that eliminated within- and across-trial variance. First, each trial, aligned at voice onset, was normalized by subtracting the participant's *F*1 unperturbed response trend (average *F*1 response in the unperturbed trials, normalized at voice onset such that *F*1(t = 0) = 0 Hz). This was done to reduce the within-trial variations in *F*1 responses of each participant. Second, each trial was then aligned at perturbation onset and normalized to the first 50 ms of the post perturbation onset data—a time before subjects could detect and begin responding to the perturbation (Houde and Nagarajan, 2011; Jenkins *et al.*, 2010; Poeppel and Hickok, 2015). This was done to reduce across-trials variations in *F*1 responses. Third, each trial was smoothed by averaging the *F*1 responses within non-overlapping 25 ms windows. This was done to reduce the formant tracking variations across frames. Finally, the trials from sessions 1, 3, and 5 were grouped together by conditions and averaged to obtain a *F*1 response time-course for each participant. The average and standard error of the *F*1 response time-courses were then calculated across participants.

The group onset response latency was calculated as the time point where the response exceeded two standard deviations from the mean of the onset response data, which are the responses in the first 50 ms after perturbation onset within perturbation conditions of the same type. For example, the group onset response latency for the −200 Hz mid-utterance perturbation condition was calculated as the time point where the response to the –200 mid-utterance perturbation exceeded two standard deviations from the mean of the first 50 ms responses in all (+200, −200, +50, and −50 Hz) mid-utterance perturbation conditions. Using the first 50 ms responses across all perturbations within the same type was done to reduce noise in the baseline data and thus increase the sensitivity of detecting the response onset latency. To obtain the distribution of peak compensation response percentages for each of the eight formant perturbation conditions, we first calculated each participant's peak response by averaging their normalized *F*1 time-course response in a 200 ms time window around the group peak response latency for each condition. The calculated peak responses were then converted into compensation percentages by using the formula Peak  Normalized  Response in Hz/Perturbation Magnitude in Hz×100%×compMult where compMult (compensation multiplier) was –1 for positive perturbations and +1 for negative perturbations, in order to make all compensatory responses positive regardless of the sign of the perturbation. Violin plots were created using the peak compensation response percentages, and a one-sample two-tailed t-test for each condition was calculated to test for significance different from zero. A linear mixed effects (LME) model was run in sas 9.4 (sas Institute Inc., Cary, NC) using the proc mixed procedure to evaluate the main effect of perturbation magnitude (50 vs 200 Hz), direction (positive vs negative), and type of perturbation onset (mid- vs whole-utterance) on the individual peak percent compensation responses with participant as a random factor. To account for covariation of participant's age and baseline *F*1 production (non-normalized *F*1 production at perturbation onset, calculated for each perturbation condition), we added these factors as nuisance covariates to the LME model.

#### 2. Relationship between responses to unpredictable perturbations at different onsets

To evaluate the relationship between responses to unpredictable perturbations at mid-utterance and at utterance onset, we compared the peak compensation response percentages for the unpredictable mid-utterance perturbations and to unpredictable whole-utterance perturbations. Scatterplots were created for the responses to each of the four perturbation values (+50, −50, +200, and −200 Hz) as well as for responses to all perturbation values combined. For each scatterplot, we fitted a linear mixed-effects model using the fitlme function in matlab. The formula used was for a random intercept model with a fixed slope, whole ∼ 1+mid+(1|participants), where whole and mid represent peak compensation response percentages for unpredictable whole- and mid-utterance perturbations, respectively.

#### 3. Sensorimotor adaptation responses to consistent, predictable formant perturbations

To calculate the sensorimotor adaptation response over the course of the trials in the adaptation sessions, trials from sessions 2 and 4 were analyzed by averaging the first 75 ms of *F*1 data points in each trial. The first 75 ms of *F*1 data were examined to isolate the initial feedforward adaptation responses from subsequent feedback-based within-trial responses (75 ms is a time before subjects could detect and begin any within-trial online compensatory response to the perturbation) (Houde and Nagarajan, 2011; Jenkins *et al.*, 2010; Poeppel and Hickok, 2015). Each participant's *F*1 response across trials was then normalized using their average *F*1 responses in the baseline phase (first 30 “head” trials) to highlight each participants' *F*1 sensorimotor adaptation response changes and smoothed by averaging this *F*1 normalized response within non-overlapping five-trial windows to reduce formant tracking variations across trials. The mean and standard error of the *F*1 across-trial sensorimotor adaptation response were then calculated across participants.

To obtain the distribution of sensorimotor adaptation response percentages for +200 and −200 Hz *F*1 perturbations, we first obtained sensorimotor adaptation responses by averaging each participant's normalized *F*1 trial-course response in last 15 “head” trials of the hold phase (trials 76–90). The calculated sensorimotor adaptation responses were then similarly converted into compensation percentages as described in the unpredictable perturbation section above. Violin plots were created using these sensorimotor adaptation response percentages, and a one-sample two-tailed t-test for each condition was calculated to test for significance different from zero. A LME model was run in sas to evaluate the main effect of perturbation direction (positive vs negative) on the sensorimotor adaptation response percentages with participant as a random factor. To account for covariation of participant's age and baseline *F*1 production (average first 75 ms *F*1 production in all trials of the baseline phase, calculated for each perturbation direction), we added these factors as nuisance covariates to the LME model.

#### 4. Relationship between responses to unpredictable and consistent, predictable perturbations

To evaluate the relationship between responses to unpredictable perturbations and to consistent, predictable perturbations, we compared the sensorimotor adaptation responses to consistent, predictable perturbations to the peak compensation responses to both types of unpredictable perturbations. Scatterplots were created for the comparisons of sensorimotor adaptation responses to responses to each of the unpredictable perturbation types (mid- and whole utterance). For each scatterplot, we fitted a linear mixed-effects model using the formula adapt ∼ 1+unpredict+(1|participants), where adapt represents sensorimotor adaptation response percentages and unpredict represents peak compensation response percentages for unpredictable (either mid- or whole utterance) perturbations.

#### 5. Within-trial responses in adaptation experiments

We investigated whether within-trial compensation drives sensorimotor adaptation by examining the within-trial responses in the different phases of the adaptation experiments. To do this, we selected a subset of participants who showed both a within-trial online compensation response to the unpredictable perturbations as well as a sensorimotor adaptation response to consistent, predictable perturbations. There were 15 participants who positively compensated to the +200 Hz unpredictable whole-utterance perturbations and positively adapted to the +200 Hz consistent, predictable perturbations. There were 12 participants who positively compensated to the −200 Hz unpredictable whole-utterance perturbations and positively adapted to the −200 Hz consistent, predictable perturbations.

To obtain the within-trial responses in the different phases of the adaptation experiments, each selected participant's within-trial *F*1 response time-courses in “head” trials were averaged in each of the following analysis phases: late baseline phase (last 15 “head” trials in baseline phase, trials 16–30), early adaptation phase (first 15 “head” trials in hold phase, trials 31–45), late adaptation phase (last 15 “head” trials in hold phase, trials 76–90), and late washout phase (last 15 “head” trials in washout phase, trials 106–120). The individual averaged within-trial *F*1 response time-courses were then normalized by subtracting the individual's average *F*1 time-course response in the baseline phase (first 30 trials). This was done reduce the within-trial variations in *F*1 responses of each participant. The average and standard error of the time-course within-trial *F*1 responses in the different phases of the adaptation experiments were then calculated across all selected participants.

To highlight the change in responses in the initial feedforward responses across the different phases, we plotted boxplots showing the distribution of the selected participants' response onsets (0–75 ms) in terms of percent compensation for each analysis phase in the adaptation experiment [Figs. 6(c) and 6(d)]. One-sample t-test for each boxplot distribution was calculated to test for significance different from zero. Additionally, to highlight the change in within-trial responses, we plotted pairwise scatterplot overlaid over boxplots showing the distribution of the selected participants' responses at onset (“O,” 0–75ms) and at mid-utterance (“M,” 600–800 ms) in terms of percent compensation for each phase in the adaptation experiment [Figs. 6(e) and 6(f)]. A LME model was run in sas to evaluate the main effect of within-trial time window (0–75 vs 600–800 ms), adaptation analysis phase (late baseline, early adaptation, late adaptation and late washout), and perturbation direction (+200 vs −200 Hz) on the individual within-trial percent compensation responses with participant as a random factor.

#### 6. Evaluating session order effects

In our experimental design, two out of the three sessions consisting of unpredictable formant perturbations (i.e., sessions 3 and 5) followed sessions with adaptation experiments (sessions 2 and 4). Given that previous studies have shown short-term changes in speech motor control following adaptation experiments (Ito *et al.*, 2016; Shiller *et al.*, 2009), this experimental structure may have had carryover effects from the consistent, predictable perturbation sessions to the following unpredictable perturbation sessions. To examine whether any such carryover effects took place in our study, we performed two analyses on *F*1 production in the unpredictable perturbation sessions. The first analysis was done to investigate whether participant's baseline *F*1 production shifted across the three sessions. To do this, we examined the first 50 ms of the *F*1 formant tracks in each trial in sessions 1, 3, and 5. This time window was selected to avoid including any possible within-trial responses to the unpredictable perturbations. An analysis of variance (ANOVA) was then performed on the median baseline *F*1 production to evaluate the effect of session order with participants as a random factor. A second analysis was done to investigate whether participant's peak compensation response percentages shifted across the three sessions. To do this, we calculated the peak percent compensation response percentages for each perturbation condition, similar to what was done above, but separately for each session normalized response average. A LME model was then run to evaluate the main effect of session order (sessions 1, 3, and 5), perturbation magnitude (50 vs 200 Hz), perturbation direction (positive vs negative) and type of perturbation onset (mid- vs whole-utterance) on the peak percent compensation response percentages with participant as a random factor.

## III. RESULTS

### A. Participants compensated to unpredictable mid- and whole-utterance formant perturbations

Participants exhibited within-trial online compensation not only for *F*1 perturbations applied unpredictably for the whole-utterance [Fig. 2(b)], but also for *F*1 perturbations applied unpredictably at mid-utterance [Fig. 2(a)]. Response onset latencies for −50, +50, −200, and +200 Hz perturbations are shown in Table I. The distributions of peak compensation responses are shown as violin plots in Figs. 2(c) (mid-utterance) and 2(d) (whole utterance), and significance levels, when compared to zero, are shown in Table I. Responses to mid- and whole-utterance perturbations were significant compared to zero with the exception of responses to −50 Hz mid-utterance perturbations. A LME model of peak compensation response percentages showed significant main effects for perturbation magnitude [200 vs 50 Hz: *F*(1,146) = 8.32, *p* = 4.50 × 10^−3^) and direction (positive vs negative: *F*(1,146) = 5.10, *p* = 0.03], but not for type of perturbation [mid- vs whole-utterance: *F*(1,146) = 3.72, *p* = 0.06]. There are no significant interaction between perturbation magnitude and direction [*F*(1,146) = 4.52, *p* = 0.11], perturbation magnitude and type of perturbation [*F*(1,146) = 0.72, *p* = 0.40], perturbation direction, and type of perturbation [*F*(1,146) = 0.02, *p* = 0.90] and between perturbation magnitude, direction, and type of perturbation [*F*(1,146) = 0, *p* = 0.99]. No significant effects for the covariates of age or baseline *F*1 production was found [*F*(1,146) = 0.23, *p* = 0.63 and *F*(1,146) = 1.51, *p* = 0.22, respectively]. Overall, participants significantly compensated for both unpredictable mid- and whole-utterance *F*1 perturbations.

### B. Within-trial compensations for unpredictable mid- and whole utterance *F*1 perturbations were significantly correlated

**FIG. 3.**
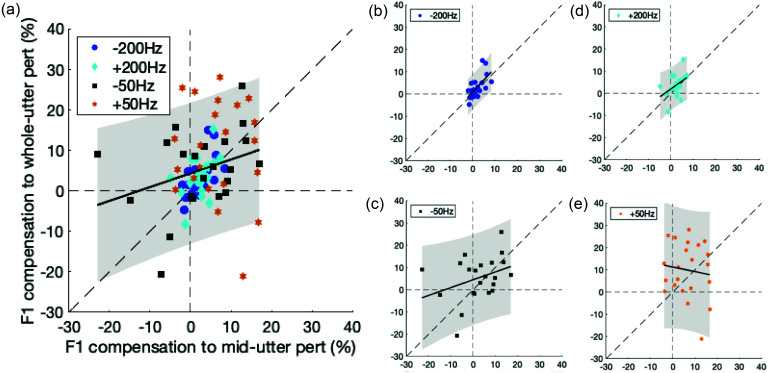
**Compensatory responses to mid-utterance perturbations are correlated with responses to whole utterance perturbations.** Scatterplot of peak compensation responses to transient mid-utterance and to whole-utterance formant perturbations for (a) all conditions and separately for (b–e) −200, −50, +200, and +50 Hz perturbation conditions, respectively. Responses to −200 Hz perturbations are indicated with circles, +200 Hz perturbations with diamonds, −50 Hz perturbations with squares, and +50 Hz perturbations with hexagrams. The slope and 95% confidence interval of the correlations are indicated by the solid black line and gray shaded area, respectively. Dashed lines represent the coordinate axes and the diagonal with slope = 1.

We found a positive correlation between responses to unpredictable mid-utterance and whole-utterance perturbations. Correlation was significantly positive when all four perturbation values were aggregated with participant as a random factor [Fig. 3(a): Regression coefficient estimate= 0.34, *p* = 0.02]. When the correlation analysis was separately performed for each perturbation value, mid- and whole- utterance perturbation responses were significantly correlated only for responses to +200 and −200 Hz perturbations [Fig. 3(b): Regression coefficient estimate = 1.08, *p* = 6.23 × 10^−4^ for −200 Hz perturbations; Fig. 3(c): Regression coefficient estimate = 0.35, *p* = 0.10 for −50 Hz perturbations; Fig. 3(d): Regression coefficient estimate = 0.71, *p* = 0.03 for +200 Hz perturbations; Fig. 3(e): Regression coefficient estimate = –0.21, *p* = 0.59 for +50 Hz perturbations].

### C. Participants adapted to consistent, predictable *F*1 perturbations

**FIG. 4.**
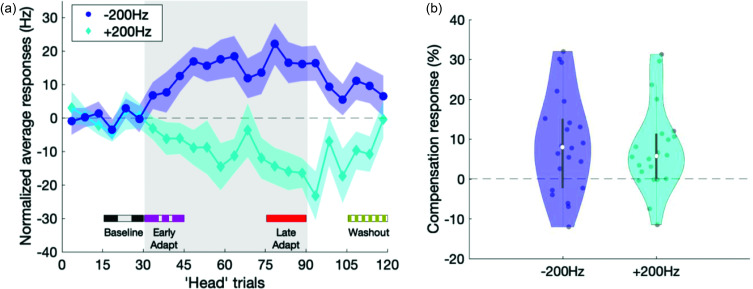
**Sensorimotor adaptation results to consistent, predictable formant perturbations**. (a) Average responses in the first 75 ms of each trial to consistent, predictable formant perturbations across trials; average over five “head” trials (points joined by solid line) and standard error (shaded colored region) are shown. Responses to −200 and +200 Hz are shown with circles and diamonds, respectively. Shaded gray region represents trials where whole-utterance perturbation was consistently applied. Colored bars at the bottom indicate phases of trials in the adaptation experiment used for the within-trial time course analysis presented in Fig. 6: baseline phase (“head” trials 16–30, dashed gray line), early adaptation phase (“head” trials 31–45, dashed-dotted magenta line), late adaptation phase (“head” trials 76–90, solid red line), and washout phase (“head” trials 106–120, dotted gold line). (b) The distributions of adaptation responses in the late adaptation phase are shown as violin plots. Table II lists the *p*-values for significant differences from zero of the sensorimotor adaptation responses.

Consistent with what has been seen in earlier studies, participants adapted their initial feedforward responses to a consistent, predictable *F*1 perturbation across trials in the adaptation experiments. Sensorimotor adaptation response was quantified here as the initial 75 ms *F*1 response in each trial that was then averaged for every five successive trials. Figure 4(a) shows the normalized sensorimotor adaptation response across trials of the adaptation experiments for +200 Hz (blue) and –200 Hz (cyan). The distributions of individual sensorimotor adaptation response percentages quantified in the late adaptation phase are shown as violin plots in Fig. 4(b) and significance levels, when compared to zero, are shown in Table II. A LME model of sensorimotor adaptation responses showed no significant main effect for perturbation direction, or for the covariates of age or baseline *F*1 production [*F*(1,20) = 0.26, *p* = 0.62; *F*(1,20) = 0.03, *p* = 0.86; and *F*(1,20) = 0.23, *p* = 0.64 for direction, age, and baseline *F*1, respectively]. These findings show that the sensorimotor adaptation response to consistent, predictable *F*1 feedback perturbations can be observed within the initial feedforward response.

**TABLE II.** Sensorimotor adaptation responses across participants (mean and SEM) were calculated from the first 75 ms of each of the last 15 “head” trials of the hold phase (late adaptation phase). T-values (two-tailed) and *p-*values indicate the significant difference from zero of the sensorimotor adaptation responses.

Perturbation value (Hz)	Adaptation responses (%) (Mean ± SEM)	T-values (DoF = 21)	Significance level (*p-*value)
−200	8.98 ± 2.63	3.41	2.63 × 10^−3^
+200	7.67 ± 2.29	3.45	3.04 × 10^−3^

### D. Online compensation for unpredictable feedback perturbations and sensorimotor adaptation to consistent, predictable feedback perturbations were not correlated

**FIG. 5.**
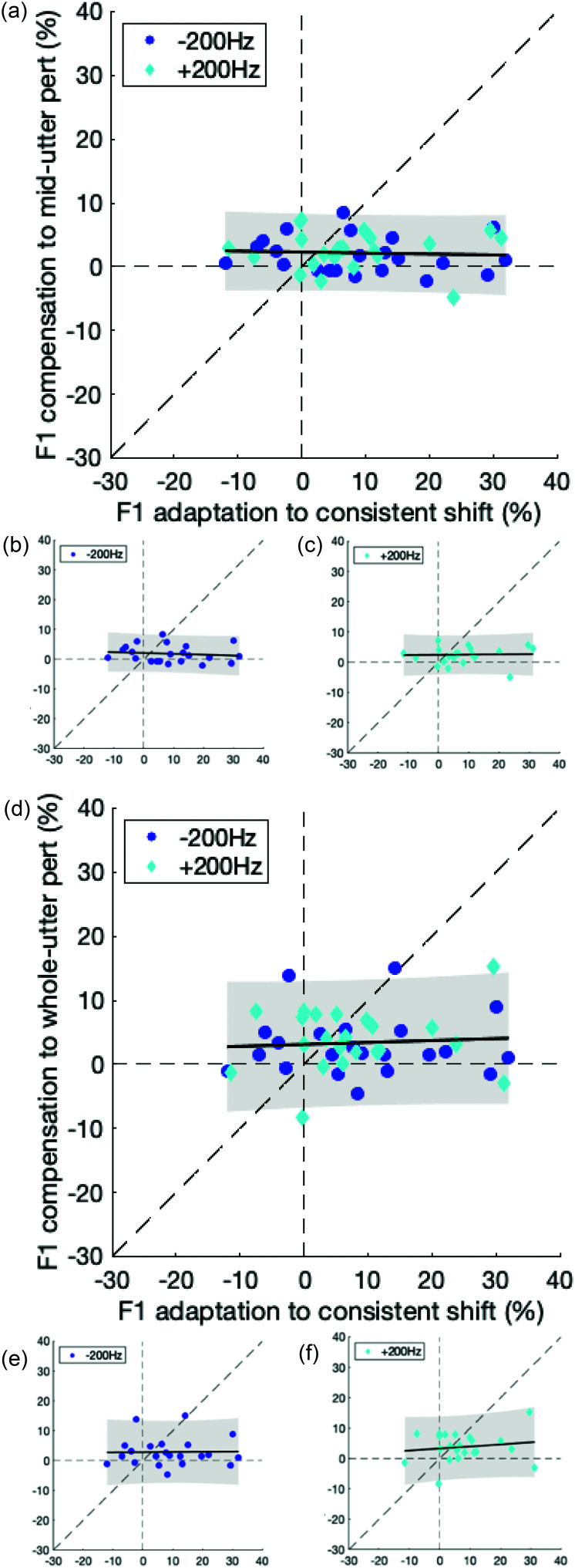
**Online formant compensation is not associated with formant sensorimotor adaptation.** Scatterplot of sensorimotor adaptation responses to consistent, predictable formant perturbations and compensation responses to unpredictable transient mid-utterance perturbations for (a) both conditions and separately for (b and c) −200 and +200 Hz perturbation conditions, respectively. Scatterplot of sensorimotor adaptation responses to consistent, predictable formant perturbations and online compensation responses to whole-utterance perturbations for (d) for both conditions and separately for (e and f) −200 and +200 Hz perturbation conditions, respectively. The slope and 95% confidence interval of the correlations are indicated by the solid black line and gray shaded area, respectively. Dashed lines represent the coordinate axes and the diagonal with slope = 1.

Responses to unpredictable *F*1 feedback perturbations (online compensation) and to consistent, predictable *F*1 feedback perturbations (sensorimotor adaptation) were generally uncorrelated (Fig. 5). Correlations between responses to mid-utterance perturbations and sensorimotor adaptation responses were all non-significant [Fig. 5(a): Regression coefficient estimate = –0.12, *p* = 0.80 for all conditions aggregated with participants as a random effect; Fig. 5(b): Regression coefficient estimate = –0.53, *p* = 0.56 for −200 Hz perturbations; and Fig. 5(c): Regression coefficient estimate = 0.09, *p* = 0.90 for +200 Hz perturbations). Similarly, correlations between responses to whole-utterance perturbations and sensorimotor adaptation responses were also non-significant [Fig. 5(d): Regression coefficient estimate = –0.12, *p* = 0.67 for all conditions aggregated with participant as a random effect; Fig. 5(e): Regression coefficient estimate = –0.04, *p* = 0.94 for −200 Hz perturbations; and Fig. 5(f): Regression coefficient estimate = 0.33, *p* = 0.48 for +200 Hz perturbations].

### E. Within-trial response dynamics in sensorimotor adaptation further revealed independence between online compensation responses and sensorimotor adaptation

**FIG. 6.**
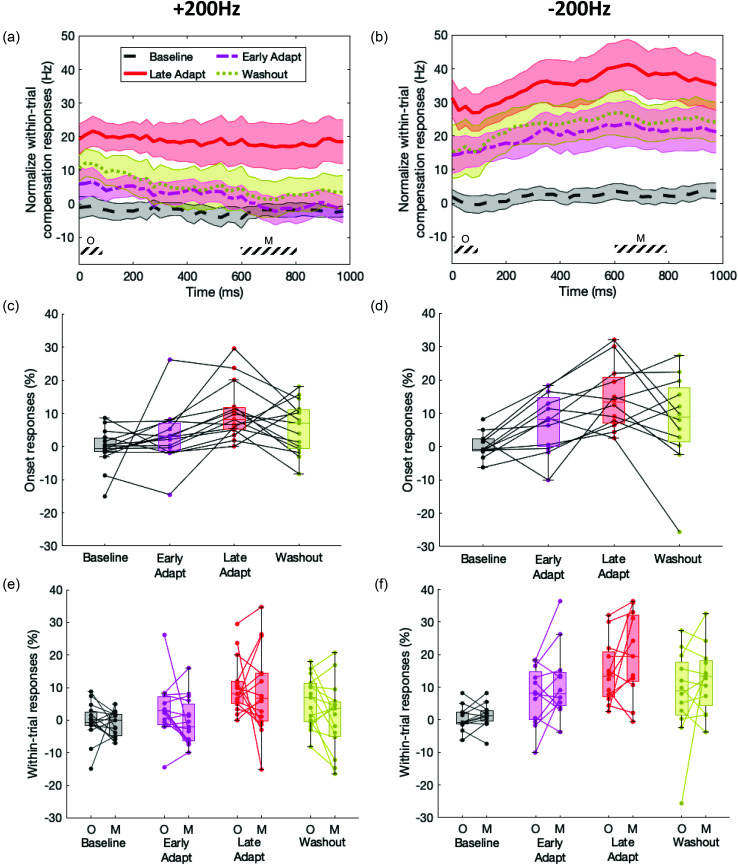
**Evolution of within-trial formant time-course across the adaptation experiment.** Normalized within-trial perturbation responses in different phases of the adaptation experiments with (a) +200 and (b) −200 Hz consistent, predictable perturbations. Mean responses (lines) and SEM (shaded colored region) are shown. The colors represent responses in different ranges of “head” trials: baseline (“head” trials 16–30, dashed gray), early adaptation (“head” trials 31–45, dashed-dotted magenta), late adaptation (“head” trials 76–90, solid red), and washout (“head” trials 106–120, dotted gold). These ranges are indicated as bars at the bottom of Fig. 4(a). The plots show both changes in initial response as well as within-trial response time course in different phases of the adaptation experiments. Shaded bars at the bottom of the plots indicate time windows for onset (“O,” 0–75 ms) and mid-utterance (“M,” 600–800 ms) within-trial compensation response. (c and d) Boxplots show Onset (0–75 ms) within-trial compensation responses distribution for +200 and −200 Hz adaptation experiment respectively. Lines connect individual subjects' responses. (e and f) Pairwise onset (“O,” 0–75 ms) and mid-utterance (“M,” 600–800ms) within-trial compensation response distribution for +200 and –200 Hz adaptation experiment, respectively. Lines connect individual subjects' responses.

We explored how the within-trial response dynamics changed over the course of the trials in the adaptation experiment. For this analysis, we examined the within-trial responses of participants who both positively compensated to whole-utterance perturbations and also positively adapted to consistent, predictable perturbations (Fig. 6). This was done to ensure that the analysis would not be biased by participants who did not show a within-trial response (in the whole-utterance perturbations). A total of 15 participants positively compensated to the +200 Hz unpredictable whole-utterance perturbations and positively adapted to the +200 Hz consistent, predictable perturbations. A total of 12 participants positively compensated to the −200 Hz unpredictable whole-utterance perturbations and positively adapted to the −200 Hz consistent, predictable perturbations. The within-trial responses shown [Figs. 6(a) and 6(b)] were normalized to each participant's within-trial response in the baseline phase (“head” trials 1–30).

This analysis showed changes even in the initial 75 ms of the average within-trial time-course of each phase of the adaptation experiment, showing evidence of sensorimotor adaptation in the initial feedforward response. The distribution across participants of the initial (0–75 ms) within-trial responses in each trial, expressed as percent compensation, are shown across phases of the adaptation experiment [Figs. 6(c) and 6(d)]. The initial 75 ms response in the −200 Hz adaptation experiment was not significantly above zero in baseline or washout phases [baseline phase: mean ± standard error of the mean (SEM) = 0.81 ± 2.19, *t*(11) = 0.37, *p* = 0.72; and washout phase: mean ± SEM = 15.96 ± 8.03, *t*(11) = 1.99, *p* = 0.07] but was significantly above zero in the early and late adaptation phases [early adaptation phase: mean ± SEM = 14.75 ± 5.12, *t*(11) = 2.88, *p* = 0.02; and late adaptation phase: mean ± SEM = 29.25 ± 5.57, *t*(11) = 5.25, *p* = 2.71 × 10^−4^]. In contrast, in the +200 Hz adaptation experiment, the initial 75 ms response was not significantly above zero in baseline and early adaptation phases [baseline phase: mean ± SEM = 0.86 ± 3.01, *t*(14) = 0.29, *p* = 0.78; and early adaptation phase: mean ± SEM = –6.22 ± 4.39, *t*(14) = –1.42, *p* = 0.18] but was significantly above in late adaptation and washout phases [late adaptation phase: mean ± SEM = –20.51 ± 4.28, *t*(14)= –4.79, *p* = 2.87 × 10^−4^; and washout phase: mean ± SEM= –11.58 ± 3.97, *t*(14) = –2.91, *p* = 0.01, respectively].

Comparisons of the late (600–800 ms) within-trial responses to the initial 75 ms within-trial responses over the phases of the adaptation experiment except the baseline phase showed that the sensorimotor adaptation response occurred regardless of the existence of a within-trial compensatory response. The baseline phase was not included in this analysis because no perturbations had yet been applied in this phase, so within-trial responses were not expected. A LME model for within-trial responses showed a significant main effect of phases [early adaptation, late adaptation and washout; *F*(2,133) = 11.91, *p* < 0.0001] and perturbation direction [−200 vs +200 Hz; *F*(1,133) = 29.68, *p* < 0.0001] but not of within-trial time window [0–75 vs 600–800 ms from voice onset; *F*(1,133) = 0.25, *p* = 0.62], with a significant interaction between within-trial time window and perturbation direction [*F*(1,133) = 7.43, *p* = 7.30 × 10^−3^]. No significant interaction was found between phases and within-trial time window [*F*(2,133) = 0.17, *p* = 0.84], between phases and perturbation direction [*F*(2,133) = 0.06, *p* = 0.94], or between phases, within-trial time window, and perturbation direction [*F*(2,133) = 0.07, *p* = 0.93]. Given the significant main effect of perturbation direction, we then analyzed the within-trial responses to −200 and +200 Hz consistent, predictable perturbations separately. LME model for −200 Hz showed significant main effects of phases [*F*(2,55) = 5.42, *p* = 7.10 × 10^−3^] and within-trial time window [*F*(1,55) = 4.59, *p* = 0.04] but no significant interaction between phases and within-trial time window [*F*(2,55)= 0.03, *p* = 0.97]. LME model for +200 Hz showed a significant main effect of phase [*F*(2,70) = 7.05, *p* = 1.6 × 10^−3^], no significant main effect of within-trial time window [*F*(1,70)= 3.00, *p* = 0.09] and no significant interaction between phases and within-trial time window [*F*(2,70) = 0.25, *p* = 0.78].

### F. *F*1 production in unpredictable perturbation sessions were not affected by experimental session order

We found no significant main effect of session order on the median baseline *F*1 production across the unpredictable perturbation sessions [*F*(2,21) = 0.41, *p* = 0.67]. The second analysis was done on peak compensation responses in each of the three unpredictable perturbation sessions. Once again, we found no significant main effect of session order on peak compensation responses [*F*(1,498) = 0.02, *p* = 0.90]. We also found no significant main effect of perturbation magnitude [*F*(1,498) = 1.29, *p* = 0.26] and type of perturbation onset [*F*(1,498) = 2.87, *p* = 0.09] but found a significant main effect of perturbation direction [*F*(1,498) = 6.84, *p* = 9.2 × 10^−3^]. These findings indicate that there were no significant carryover effects from the adaptation experiments to the following unpredictable perturbation sessions.

## IV. DISCUSSION

In this study, we showed clear evidence for online auditory feedback control of formants. We observed that similar online auditory feedback compensation mechanisms are evident both at utterance onset and at mid-utterance. We also observed that auditory feedback compensation responses were not associated with sensorimotor adaptation of formants. These results have important implications for speech motor control models.

### A. Transient auditory feedback perturbations induce similar responses in control of formants, pitch and loudness

We show that speakers produce short-latency compensatory responses to transient mid-utterance perturbations of *F*1 of the auditory feedback of their speech. The magnitude of these compensatory responses was significantly affected by the perturbation magnitude, where responses to 50 Hz perturbations were larger than to 200 Hz perturbations, consistent to what has been observed in other studies of formant perturbations (Daliri *et al.*, 2020; Katseff *et al.*, 2012). The formant feedback perturbations we used had a similar time-course (abrupt with jittered onset) to the transient mid-utterance perturbations used in studies showing compensatory responses to pitch and loudness perturbations (Burnett *et al.*, 1998; Heinks-Maldonado and Houde, 2005; Keough *et al.*, 2013; Kort *et al.*, 2016; Larson *et al.*, 2001; Larson *et al.*, 1999). Although mid-utterance formant perturbations have been investigated in a previous study, in that study, the perturbation was always applied 300 ms after voice onset and was cross-faded in linearly over 500 ms (Purcell and Munhall, 2006b). Therefore, the perturbations used in the current study allowed for more direct comparison with pitch and loudness feedback perturbation responses. A striking similarity in responses to the transient mid-utterance perturbations across the speech features is the latency of response. In pitch and loudness perturbations, studies have found a response latency ranging around 150–250 ms (Burnett *et al.*, 1998; Burnett *et al.*, 1997; Heinks-Maldonado and Houde, 2005). In formant perturbations, the response latency ranges around 200–325 ms. The response onset time similarity suggests a similar timescale for the auditory feedback processing of these different speech features.

While this may be considered surprisingly fast given the greater mass of the tongue and upper vocal tract when compared to the vocal folds, a recent study done by Bakst (2017) that used ultrasound to track tongue movement during the production of varying consonant, vowel, consonant (CVC) English words found that amount of tongue movement needed to make an acoustic change can be very small. Even though the tongue is a big muscle organ, it needs to be moved only by a very small amount to achieve a compensation response. The jaw may not technically be involved/needed for this compensation response to occur. Therefore, the size of the system may appear to be not an important factor in determining the reaction time which refers to only the starting point of response, representing how responsive the system is to the motor command. Consistent with this notion, a study by Pfister *et al.* (2014) shows how larger organs like hand and foot even have a reaction time of about 300 ms.

### B. Feedback control of formants at utterance onset and at mid-utterance share a similar mechanism

We explored the possible differences in feedback control of formants at utterance onset and at mid-utterance by comparing online compensation responses to formant feedback perturbations applied for the whole utterance and transiently at mid-utterance. Previously, Hawco and Jones (2009) found a large difference (∼50% vs ∼17%) between the peak response to pitch feedback perturbations at utterance onset and at mid-utterance, and they suggested that the difference is due to different control mechanisms operating at utterance onset and at mid-utterance. In our study, we found slightly larger peak responses to formant perturbations at utterance onset (∼5%) than at mid-utterance (∼3%), and this difference was not statistically significant.

What can explain this difference in utterance onset and mid-utterance perturbation responses between pitch and formants? First, it should be noted that in the Hawco and Jones (2009) study, participants were given an external pitch reference at every trial, whereas in this study no such reference was provided. Second, other studies have shown the control of pitch may be special in its heightened reliance on auditory feedback for its control. In particular, studies of the speech of post-lingually deafened adults have shown that such deafness quickly degrades the control of pitch, while speech intelligibility can remain intact for years (Cowie and Douglas-Cowie, 1992; Houde and Nagarajan, 2015, pp. 267–298; Lane and Webster, 1991). Third, it is possible that underlying mechanisms for the control of pitch and formants are different. Previous studies have found that individual with Parkinson's disease produced significantly larger compensation responses to pitch perturbations (Chen *et al.*, 2013; Liu *et al.*, 2012) but smaller compensation responses to formant perturbations (Mollaei *et al.*, 2016). These findings may reflect differences in the control of formant and pitch, perhaps where recruitment of sensorimotor regions may be greater for the control of formants that involve a larger set of muscles and associated sources of somatosensory feedback in the vocal tract than for pitch (Bouchard *et al.*, 2013; Huang *et al.*, 2019; Mollaei *et al.*, 2016).

In addition to responses to formant perturbations at onset and at mid-utterance not being significantly different in magnitude, we further showed a strong correlation between whole utterance and mid-utterance perturbation responses, suggesting a similar mechanism governing these two responses. The idea that onset and mid-utterance formant control are governed by a similar mechanism can be explained by the SFC model (Parrell and Houde, 2019) by making assumptions about the Kalman gain. In SFC, the Kalman gain is responsible for modulating the corrective response to a mismatch between actual and expected feedback (in this case, brought upon by perturbations). Our results and those of Hawco and Jones (2009) could be explained by (1) the Kalman gain for pitch control being larger than it is for formant control, and (2) the Kalman gain being larger at utterance onset, where there is more production uncertainty than during continuation of the utterance. Therefore, our results suggest that the control mechanism throughout an utterance, whether it be at onset or at mid-utterance, is the same but potentially with varying magnitude of Kalman gain at different times (onset vs mid-utterance) and for different speech features (formants vs pitch).

### C. Online compensation and sensorimotor adaptation are governed by different control mechanisms

Many speech motor control models acknowledge the existence of two speech control mechanisms: feedback and feedforward control (Guenther, 2016; Kearney *et al.*, 2020; Parrell *et al.*, 2019). Feedback control is responsible for maintaining speech production within an utterance, which is exhibited by the online compensation responses to unpredictable perturbations. In contrast, feedforward control is responsible for generating correct motor commands for an intended speech sound; feedforward motor commands are learned from past experiences and their execution does not depend on sensory feedback during production. However, the feedforward system can learn a new speech motor mapping if it consistently detects a mismatch between the actual and expected speech feedback over a long period of time, which leads to a sensorimotor adaptation response seen in numerous studies (Houde and Jordan, 1998; Jones and Munhall, 2000; Katseff *et al.*, 2012; Purcell and Munhall, 2006a).

Despite being well-studied, we still do not have a clear understanding of how exactly the feedforward system is able to adapt to consistently perturbed feedback. As a result, models of speech differ in how feedback and feedforward control components are related. The DIVA model suggests that “…the feedforward control system constantly monitors the corrective commands generated by the feedback control system, gradually incorporating repeatedly occurring corrections into the feedforward command” (Guenther, 2016). Specifically, the DIVA model has four separate parameters governing: (1) responses to auditory feedback perturbations, (2) responses to somatosensory feedback perturbations, (3) incorporation of auditory feedback responses into the feedforward controller, and (4) incorporation of somatosensory feedback responses into the feedforward controller. A simpler three-parameter version of this model, SimpleDIVA, uses a single parameter to govern the incorporation of both auditory and somatosensory feedback responses into the feedforward controller, while retaining the two feedback control parameters (Kearney *et al.*, 2020). Thus, both DIVA and SimpleDIVA assume a close relationship between feedback and feedforward control. In contrast, the SFC model accommodates distinct mechanisms for feedback control and the learning of feedforward control, as adaptation can arise directly from sensory prediction errors rather than necessarily being derived from the incorporation of corrective movements as is assumed in DIVA (Parrell and Houde, 2019). In our study, we found three lines of evidence that the feedforward sensorimotor adaptation mechanism is distinct from the feedback control mechanism.

First, we found sensorimotor adaptation exceeded online compensation. We looked at the first 75 ms formant responses for each trial in the adaptation experiments to purely isolate the feedforward responses. Our results are comparable to previous findings (Behroozmand and Sangtian, 2018; Houde and Jordan, 1998; Katseff *et al.*, 2012; Purcell and Munhall, 2006a), where participants on average showed significant sensorimotor adaptation to the consistent, predictable perturbation. Importantly, however, even within the first 15–20 trials, we saw that the sensorimotor adaptation response grew larger than the peak online compensation response. While this can be explained by both DIVA and SFC, the maximum asymptotic sensorimotor adaptation response could have not exceeded the maximum asymptotic online compensation response in SimpleDIVA.

Second, we found that online compensation and sensorimotor adaptation responses were not significantly correlated across participants, as was also found in a previous study (Franken *et al.*, 2019). In fact, we found that some participants were able to adapt even though they did not compensate for the unpredictable perturbations and vice versa.

Third, we found that sensorimotor adaptation in the feedforward response was able to take place regardless of the within-trial response pattern. We were able to examine the dynamics of the adaptation response through a novel within-trial time-series analysis of the adaptation response. We found that responses to −200 Hz consistent, predictable perturbations showed some evidence of within-trial compensation which could also be seen in the late phase of the adaptation experiment, superposed on a feedforward adaptation response that started at the beginning of each trial. However, for responses to +200 Hz consistent, predictable perturbations, we did not find evidence for online compensation either in the early or late phases of the adaptation experiment, but we found clear evidence for the development of a feedforward sensorimotor adaptation response that started at the beginning of each trial. Together, the evidence is more consistent with the idea that online compensation and sensorimotor adaptation are driven by distinct neural mechanisms.

It is likely that what we have learned in this study is also applicable to dynamic speech. This study examined the effects of feedback perturbations on the production of static vowels, which lack the temporal dynamics found in natural speech. However, even though producing static vowels and dynamically changing speech are different speech tasks, there is no evidence that mechanisms of processing and learning from auditory feedback perturbations are different between these tasks. In fact, there is clear evidence that the feedback processing phenomena we observed here are also at work during natural dynamic speech production where both online compensation and sensorimotor adaptation have been observed (Lametti *et al.*, 2018; Liu *et al.*, 2009).

During the review process of the current manuscript, we became aware of a recent publication from Lester-Smith *et al.* (2020) that also explored the relationship between within-trial compensation responses and across-trial sensorimotor adaptation responses to *F*1 perturbations. Although they reported a significant positive correlation between within-trial compensation responses and across-trial sensorimotor adaptation responses to *F*1 perturbations, these results were acknowledged as potentially non-significant if corrections for multiple comparisons were applied, which would be consistent with our current findings. The study also differed from the current study in terms of experimental design in many ways, including prompt words, experimental session order, study population, perturbation magnitudes, and the time windows used in their data analysis. Therefore, it is difficult to directly compare the results of that study with our findings, and future studies are warranted to explore and reconcile these discrepancies.

### D. Directional asymmetry in compensation responses

An interesting observation in our current study is the effect of perturbation direction on responses to both unpredictable within-trial and consistent, predictable across-trial perturbations. We found a significant main effect of perturbation direction in the within-trial responses to unpredictable within-trial perturbations. Specifically, we found a smaller compensation response average to negative perturbations (3.12%) than to positive perturbations (5.72%). However, we did not find a significant main effect of direction in either the within-trial or across-trial responses to consistent, predictable across-trial perturbations (adaptation experiments). Previous studies have found a directional asymmetry in responses to both unpredictable within-trial and consistent, predictable across-trial perturbations (Liu *et al.*, 2011; Mitsuya *et al.*, 2015). Liu *et al.* (2011) found a directional asymmetry in responses to unpredictable transient mid-utterance pitch perturbations, where responses to negative pitch perturbations showed larger responses than to positive pitch perturbations. Mitsuya *et al.* (2015) found a directional asymmetry in responses to consistent, predictable across-trial *F*1 perturbations, where the adaptation magnitude is smaller in the negative direction when using the vowels /i/, /I/, and /ɛ/ (but not when using the vowels /æ/, /ɔ/, or /u/). Across all these studies, there does not seem to be any consistent trend in terms of how the compensation responses were affected by the perturbation direction. Furthermore, many other factors can result in the observed asymmetries in these studies, including articulatory constraints leading to tradeoffs between auditory and somatosensory feedback and perceptual nonlinearities, as well as variation in somatosensory feedback for different directions of articulatory change (Kothare *et al.*, 2020). Therefore, whether there is a consistent directional asymmetry remains unclear.

## V. CONCLUSIONS AND FUTURE DIRECTIONS

In this study, we showed that transient mid-utterance feedback perturbations induced similar responses in formants compared to what have been shown previously in pitch and loudness. This compensation to transient formant perturbations at mid-utterance was highly correlated with compensation to whole utterance formant perturbations, suggesting that these compensations are governed by a similar mechanism. We also found evidence suggesting that online compensation and sensorimotor adaptation are governed by distinct mechanisms. Further studies need to be performed to investigate the underlying neural bases of this mechanistic difference between sensorimotor adaptation of the feedforward control and online compensation in feedback control.

## Footnotes

^1^ See supplementary material at https://www.scitation.org/doi/suppl/10.1121/10.0003440 for table listing the number of trials in each unpredictable perturbation condition.
